# Speculum versus digital insertion of Foley catheter for induction of labor in Nulliparas with unripe cervix: a randomized controlled trial

**DOI:** 10.1186/s12884-020-03029-0

**Published:** 2020-05-29

**Authors:** Hang Min Chia, Peng Chiong Tan, Sze Ping Tan, Mukhri Hamdan, Siti Zawiah Omar

**Affiliations:** 1 Department of Obstetrics and Gynaecology, Faculty of Medicine, University of Malaya, Lembah Pantai, 50603 Kuala Lumpur, Malaysia; 2 King’s College London, The Strand, London, WC2R 2LS UK

**Keywords:** Induction of labor, Foley catheter, Nulliparas, Speculum

## Abstract

**Background:**

Induction of labor (IoL) is an increasingly common obstetric procedure. Foley catheter IoL is recommended by WHO. It is associated with the lowest rate of uterine hyperstimulation syndrome and similar duration to delivery and vaginal delivery rate compared to other methods. Insertion is typically via speculum but digital insertion has been reported to be faster, better tolerated and with similar universal insertion success compared to speculum insertion in a mixed population of nulliparas and multiparas. Transcervical procedure is more challenging in nulliparas and when the cervix is unripe. We evaluated the ease and tolerability of digital compared to speculum insertion of Foley catheter for induction of labor in nulliparas with unripe cervixes.

**Methods:**

A randomized trial was performed in a university hospital in Malaysia. Participants were nulliparas at term with unripe cervixes (Bishop Score ≤ 5) admitted for IoL who were randomized to digital or speculum-aided transcervical Foley catheter insertion in lithotomy position. Primary outcomes were insertion duration, pain score [11-point Visual Numerical Rating Scale (VNRS)], and failure. All primary outcomes were recorded after the first insertion.

**Results:**

Data from 86 participants were analysed. Insertion duration (with standard deviation) was 2.72 ± 1.85 vs. 2.25 ± 0.55 min *p* = 0.12, pain score (VNRS) median [interquartile range] 3.5 [2–5] vs. 3 [2–5] *p* = 0.72 and failure 2/42 (5%) vs. 0/44 (0%) *p* = 0.24 for digital vs speculum respectively. There was no significant difference found between the two groups for all three primary outcomes. Induction to delivery 30.7 ± 9.4 vs 29.6 ± 11.5 h *p* = 0.64, Cesarean section 25/60 (64%) vs 28/64 (60%) RR 0.9 95% CI *p* = 0.7 and maternal satisfaction VNRS score with the birth process 7 [IQR 6–8] vs 7 [7–8] *p* = 0.97 for digital vs. speculum arms respectively. Other labor, delivery and neonatal secondary outcomes were not significantly different.

**Conclusion:**

Digital and speculum insertion in nulliparas with unripe cervixes had similar insertion performance. As digital insertion required less equipment and consumables, it could be the preferred insertion method for the equally adept and the insertion technique to train towards.

**Trial registration:**

This trial was registered with ISRCTN registration number 13804902 on 15 November 2017.

## Background

The 2015–2016 maternity statistics for NHS England reports an induction of labor (IoL) rate of 27.9% [1]. Following the publication of the ARRIVE trial [2], the future trend of IoL rate can be expected to rise in nulliparas.

A 2016 network meta-analysis finds that no method of IoL demonstrated overall superiority when considering vaginal delivery in 24 h, uterine hyperstimulation syndrome and Cesarean delivery rate; the use of a Foley catheter was associated with the lowest rate of hyperstimulation syndrome [3]. World Health Organization recommends Foley catheter as a primary method of IoL [4]. The Foley catheter is a widely available, low cost medical device [5].

There is very sparse data on catheter insertion technique: in the 47 full trial reports we managed to obtain of the 77 Foley catheter IoL trials we identified in a July 1, 2017 PubMed search, where insertion method was specified, 40/47 (85.1%) specified exclusive speculum insertion, 6/47 (12.8%) permitted either speculum or digital insertion, and only 1/47 (2.1%) used digital insertion exclusively (Appendix S1). A solitary trial with 21 participants in each arm that comprised women of mixed parity reported that digital insertion was faster, better tolerated and with identical 100% insertion success when compared to speculum insertion [6]. In contrast, a recent study in our centre of nulliparas women that underwent IoL, procedure related pain score is highest with the Bishop score obtained digitally, followed by speculum aided collection of cervical secretions and lowest with transvaginal ultrasound to measure cervical length at the same pre-induction setting [7].

In a 2012 report from 19 hospitals across the USA, IoL at term with the use of prostaglandin in unripe nulliparas has a vaginal delivery rate of 56.8–58%, whilst in unripe multiparas the vaginal delivery rate is 83.7–87.7% demonstrating the challenge in unripe nulliparas IoL [8]. In the office hysteroscopy setting, pain occurs twice as often, failure rate is higher and cervical ripening or dilatation more likely to be needed in nulliparas compared to paras women [9, 10] highlighting the challenge in nulliparas with transcervical procedures.

We sought to evaluate in a powered trial the ease and tolerability of digital compared to speculum insertion of the Foley catheter for IoL in nulliparas with unripe cervixes.

## Methods

This trial was approved by Medical Ethics Committee of University Malaya Medical Centre (Approval: 25 October 2017, reference number 2017104–5636) and registered in ISRCTN (10.1186/ISRCTN13804902, 15 November 2017) prior to trial enrolment. This study was conducted in accordance with the Declaration of Helsinki on human experimentation in University Malaya Medical Centre with first recruitment on 29 December 2017 and the last participant was discharged following delivery on 26 May 2018. The study adhered to CONSORT guidelines.

### Participants

Women admitted for IoL in our unit at University Malaya Medical Centre, Kuala Lumpur, Malaysia were assessed for eligibility through scrutinizing their medical records by care providers or co-investigator HMC. Inclusion criteria were nulliparity (no prior pregnancy > 20 weeks), unripe cervix (Bishop score ≤ 5), term gestation (≥ 37 weeks of gestation with ultrasound scan verification ≤22 weeks), ≥ 18 years old, singleton pregnancy, intact membranes, cephalic presentation and reassuring pre-induction fetal heart rate tracing. Exclusion criteria included prior use of an induction agent, suspicion of chorioamnionitis or clinical genital tract infection, known gross fetal anomaly, latex allergy and inability to consent or language difficulty.

### Recruitment and randomization

Women who fulfilled initial eligibility criteria were approached by or referred to HMC for trial participation. They were provided with the Patient Information Sheet and any verbal queries were answered by the recruiting HMC. Women with Bishop Score > 5/13 were excluded. Participants were asked on their preference of Foley catheter insertion method prior to randomization. Written informed consent was obtained from all trial participants.

Randomization to digital or speculum-aided Foley catheter insertion was by the opening of the lowest numbered sealed opaque envelope remaining. The numbered envelopes were allocated in strict sequence to recruitment order and an inventory was taken of unallocated or discarded envelopes. Randomization sequence was generated in random blocks of 4 or 8 with further within block randomization (1 to 1 ratio) by a co-investigator (PCT) not involved in recruitment.

### Interventions

HMC performed the allocated interventions. HMC was a final year trainee in a 4-year specialist training program with experience of a few hundred cases of digital Foley catheter insertion for IoL.

16F silicone-coated latex Foley catheters were used in this study instead of the 18F gauge catheter used by Jonsson [6] as this was the most commonly used bore according to our literature review (Appendix S1) and it was felt that a smaller bore may pass more easily through the nulliparas unripe cervix. The silicon-coated latex catheter is the standard Foley catheter make supplied to our hospital for bladder catheterization that was re-purposed for cervical ripening. The silicon coating is incorporated to ease insertion and maintain flexibility of the underlying latex. Foley catheters of this make are much cheaper than pure silicon catheters. The Foley catheters were purchased from a commercial hospital supplies purveyor at a cost of RM3.8 (EUR 0.82) each.

In both trial arms, participants were placed in lithotomy position in stirrups for their catheter insertion. Equipment needed for the insertion was tested before insertion.

In the digital arm, catheter tip was guided through the external cervical os to about 4 cm beyond the os by the operator’s gloved hand and fingers with the aid of a water-soluble lubricant.

In the speculum arm, a sterile Cusco speculum covered with water-soluble lubricant was inserted into the vagina to visualize the cervix, a sponge forceps were used to grasp the catheter about 4 cm from the lubricated balloon tip and the tip was pushed through the external os to about 4 cm.

The balloon was then inflated with 60 ml of sterile water [11, 12] by a research assistant. The catheter was gently retracted till resistance was felt. The fingers were then withdrawn or speculum removed. The external end of the catheter was taped without tension [13] to the medial aspect of the woman’s thigh.

Time of start of the insertion process was recorded (to the minute) using a cell phone’s synchronized clock. Using a digital stopwatch operated by a research assistant, insertion time began when the operator’s finger or speculum entered the vagina and ended at the removal of fingers or speculum. Insertion interval was recorded to the nearest second.

Participant procedure related pain was assessed using a visual numerical rating scale (VNRS: 0 no pain to 10 most severe pain imaginable) immediately after the first catheter insertion attempt. A pain score of 10 was automatically assigned if the insertion failed, to penalize failure. Participants’ preference of insertion technique for future catheter insertion was recorded following the first insertion attempt.

Failure was defined as an insertion that was not achieved within 6 min (95 centile upper limit from Jonsson [6]), request by the participant to stop or the procedure abandoned by investigator before 6 min allocated time. If an insertion failed or was abandoned, an arbitrary insertion duration of 10 min would be assigned to penalize failure. The insertion attempt was abandoned after 6 min unless imminent success was anticipated.

In cases of insertion failure, participant was counselled for a cross-over attempt. If the cross-over failed or declined, bolus vaginal dinoprostone would be offered.

### Blinding

Masking of the investigator and participants to their intervention was deemed unfeasible.

### Post intervention care

If the catheter was not expelled, it would be removed after 24 h. The catheter might be removed earlier if it was not tolerated, vaginal hemorrhage occurred or membranes ruptured spontaneously. The time (to the minute) of balloon expulsion/removal was recorded. Subsequent management of IoL was conducted according to our centre’s IoL protocol: amniotomy was performed when feasible (typically cervical dilation of > 2 cm with station ≤ − 2 cm) followed by titrated oxytocin infusion. If ripening was not achieved at 24 h, bolus vaginal dinoprostone IoL was used after maternal and fetal wellbeing were ascertained. Intrapartum care was per our centre’s labor care protocol and delivery decisions made by the attendant care provider.

Within 24 h of delivery, participant was asked to score her satisfaction with birth care experience from start of Foley catheter insertion to birth using VNRS, 0 totally dissatisfied to 10 fully satisfied. Labor and birth data were retrieved from hospital records.

### Outcomes measures

Primary outcomes were 1) insertion duration, 2) insertion related pain and 3) insertion failure (as defined above).

Secondary outcomes included participants’ preference of insertion method (assessed after insertion), use of additional method(s) for cervical ripening, insertion-to-balloon expulsion or removal interval, epidural analgesia and oxytocin (for IoL or augmentation) usage, induction to delivery interval, mode of delivery, indication for operative delivery, delivery blood loss, fever (single for more readings of temperature > 38 °C intrapartum to day 1 postpartum), inpatient diagnosis of chorioamnionitis or endometritis, maternal satisfaction with their care from intervention to birth and neonatal outcomes such as Apgar scores, neonatal admission, birth weight and umbilical cord blood pH.

### Sample size calculation

Using significant data from Jonsson’s [6], sample size was calculated for our trial’s 2 primary outcomes a) insertion duration and b) procedure related pain score (VNRS 0 to 10) and the third outcome c) insertion failure (first attempt) rate was surmised. The insertion duration derived from Jonsson’s [6] were 2 ± 1.11 min and 3 ± 1.85 min [14]: applying alpha 0.05 and power 90%, using t test, 40 participants are required in each arm for a powered study. Jonsson’s [6] found a difference in median pain score of 2 (visual analog scale); for this trial’s sample size calculation, a more conservative difference in mean pain score of 1.5 (instead of 2) was applied, pain score standard deviation of 2 assumed, alpha 0.05, power 90%, 38 participants are required in each arm. Assuming insertion success rates of 90% vs 60%, alpha 0.05, power 90% utilizing the Chi Square test, 42 participants are needed in each arm. We planned to recruit 84 women.

### Statistical analysis

Data were transcribed into SPSS (version 23, IBM). Analysis performed using Student t test for comparison of means for continuous data, Chi square test for categorical data sets and Mann-Whitney U test for ordinal data. All tests were two sided. *P* < 0.05 was set as the level of significance.

## Results

**Fig. 1:**
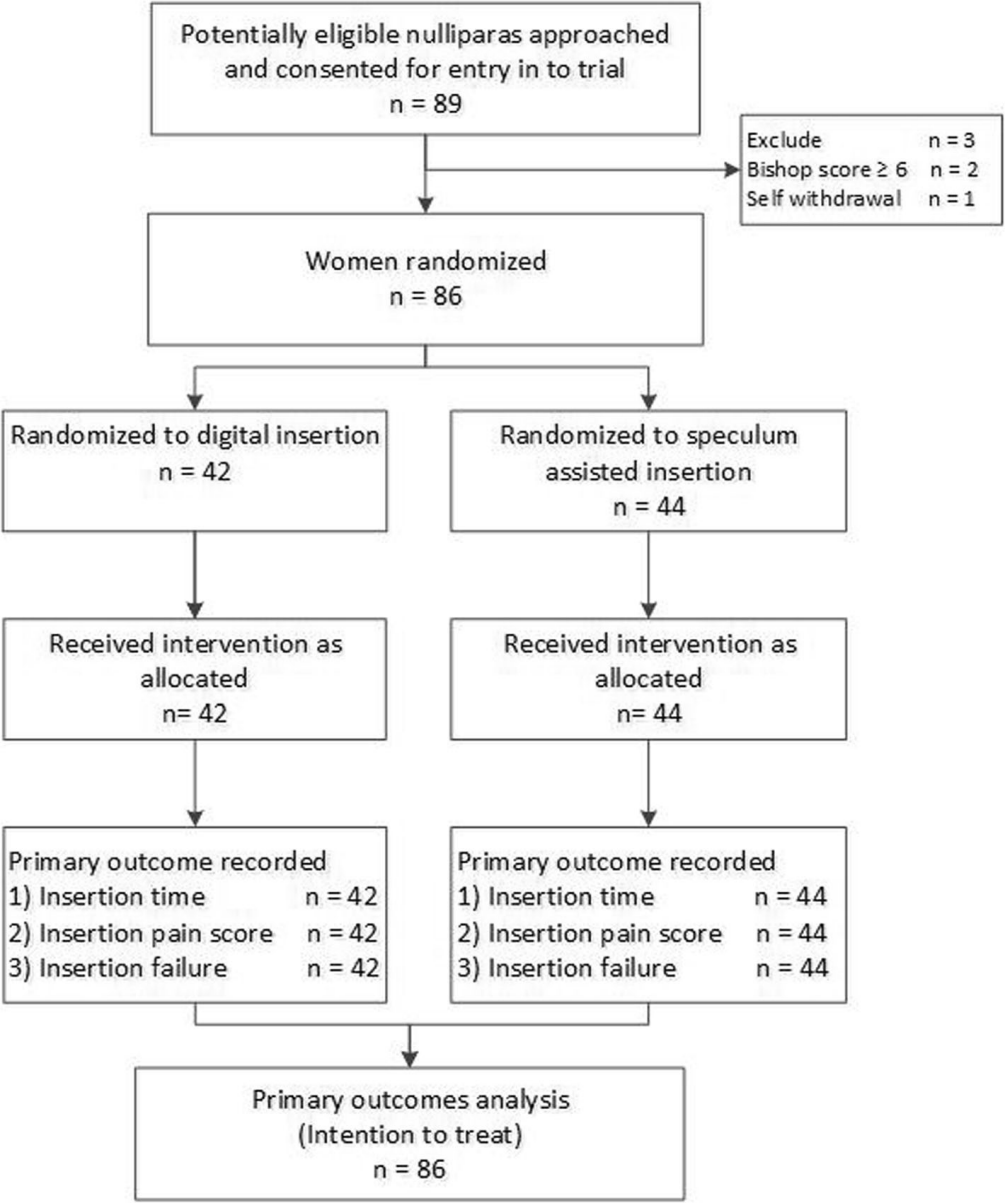
Recruitment flow chart of a randomized trial of speculum assisted compared to digital insertion of the Foley catheter for labor induction in nulliparas

During the study period, 89 nulliparas women admitted for IoL were screened eligible for enrolment and consented; 3 were later excluded before randomization (two Bishop Score > 5 and one withdrew). Eighty-six women (42 to digital and 44 to speculum insertion) were randomized and received their allocated intervention (Fig. 1). Recruitment stopped after our target sample size of 84 was exceeded.

Table 1 shows the participants’ characteristics stratified according to their random allocation. Participants in both arms had similar characteristics. Approximately 80% in each arm expressed no preference (probably reflecting their naivety to the process) and about 10% expressed preference for digital or speculum insertion, similarly in both arms.

**Table 1:** Characteristics of nulliparas with unripe cervixes after randomization to speculum or digital insertion of the Foley catheter for labor induction

	Speculum insertion (*n* = 44)	Digital insertion (*n* = 42)
Age (years)	29.3 ± 4.3	29.1 ± 3.8
*Occupation*		
Employed	41 (93)	36 (86)
Others^a^	3 (7)	6 (14)
Gestational age (weeks)	38.4 ± 1.1	38.8 ± 1.3
Height (m)	156.4 ± 5.2	157.6 ± 6.2
Body mass index (BMI)	29.8 ± 6.1	30.0 ± 6.4
*Ethnicity*		
Malay	24 (55)	23 (55)
Chinese	7 (16)	7 (17)
Indian	9 (21)	7 (17)
Other	4 (9)	5 (12)
*Indications for labor induction*		
Non-reassuring fetal status^b^	21 (48)	24 (57)
Diabetes in pregnancy	11 (25)	12 (29)
Miscellaneous^c^	12 (27)	6 (14)
Bishop score	2 [1.25–4]	3 [2–4]
*Questionnaire response to preference of insertion prior to randomisation*		
Prefers digital	6 (14)	5 (12)
Prefers speculum	4 (9)	3 (7)
No preference	34 (77)	34 (81)

Data represented as mean ± standard deviation, number (%) and median [interquartile range]

^a^Other are homemakers and students (there was no person seeking paid employment). Speculum insertion homemakers *n* = 1, students *n* = 2 while digital insertion homemakers *n* = 6

^b^Non reassuring fetal status includes suspected fetal growth restriction, small for gestational age, reported reduced fetal movement, oligohydramnios, non-reassuring fetal Doppler profiles

^c^Miscellaneous includes large for gestation/macrosomia, pregnancy induced hypertension/ preeclampsia, prolonged pregnancy, systemic lupus erythematosus, heart disease and prolonged latent phase

Primary outcomes are presented in Table 2. Of the 3 pre-specified primary outcomes, first attempt insertion duration mean (± standard deviation) 2.72 ± 1.85 vs 2.25 ± 0.55 min *p* = 0.12, pain (VNRS) median [interquartile range] 3.5 [2–5] vs 3 [2–5] *p* = 0.72 and failure 2/42 (5%; 95% CI 0.5–16.7%) vs 0/44 (0, 95% CI 0.0–9.6%) *p* = 0.24 for digital vs speculum insertion respectively were not significantly different.

**Table 2:** Primary outcomes after randomzation to speculum or digital insertion of Foley catheter for the induction of labor in nulliparas with unripe cervixes

Primary outcomes	Digital insertion (*n* = 42)	Speculum insertion (*n* = 44)	*P* value
Insertion duration (minutes)	2.72 ± 1.85	2.25 ± 0.55	0.12^a^
Failure of insertion	2 (5)	0 (0)	0.24^b^
Procedure related pain score	3.5 [2–5]	3 [2–5]	0.72^c^

Data are represented as mean ± standard deviation, number (%) or median [interquartile range]. ^a^ represents analyses by *t* test for means, ^b^ represents Fisher Exact test for categorical data and ^c^ represents Mann-Whitney *U* test for ordinal data

Both women who had failed digital insertion (one at the request of participant and another abandoned by investigator after 4 min) accepted the cross-over to speculum aided insertions which were successful. There were no major harms noted in our trial of insertion related hemorrhage, uterine perforation or feta-neonatal injury.

All downstream secondary outcomes were closely similar across the trial arms (Table 3). Fidelity to allocated insertion method if participants were to require cervical ripening and IoL in a future pregnancy were 29/42 (69%) vs 32/44 (73%) RR 0.9 95% CI 0.9 (0.7–1.2) *p* = 0.71 for digital vs speculum arms respectively, fairly high rates in both arms plausibly reflecting broad acceptability for either intervention and a stark contrast to pre-randomization when approximately 80% in each arm expressed no preference. Induction to delivery interval, Cesarean section rate (the leading indication being failure of labor to progress) and maternal satisfaction with the birth process were similar. Apgar score at 5 min, poor Apgar score (≤ 4 in at 1 min or ≤ 6 at 5 min), mean birth weight, mean umbilical cord artery pH and base excess and neonatal admission rate were also similar.

**Table 3:** Secondary outcomes after randomization to speculum or digital insertion of Foley catheter for the induction of labour in nulliparous women with unripe cervixes

Secondary outcomes	Digital (*n* = 42)	Speculum (*n* = 44)	RR (95% CI)	P value
Fidelity to allocated insertion technique	29 (69)	32 (73)	0.9 (0.7–1.2)	0.71**
Use of additional method(s) for ripening	3 (7)	1 (2)	3.1 (0.3–29.0)	0.28**
Insertion-to-balloon expulsion or removal interval (hours)	17.0 ± 5.7	15.7 ± 7.1		0.34*
Epidural analgesia	8 (19)	7 (16)	1.2 (0.5–3.0)	0.70**
Oxytocin for labor	34 (81)	36 (82)	1.0 (0.8–1.2)	0.92**
Induction to delivery interval (hours)	30.7 ± 9.4	29.6 ± 11.5		0.64*
*Mode of delivery*				0.285**
Spontaneous vaginal	15 (36)	12 (27)		
Operative vaginal^a^	2 (5)	4 (9)		
Cesarean sections	25 (60)	28 (64)	0.9 (0.7–1.3)	0.7**
*Indication for operative vaginal*				0.22
Non-reassuring fetal status	0 (0)	2 (50)		
Failure to progress	2 (100)	2 (50)		
*Indications for Cesarean delivery*			1.1 (0.5–2.8)	0.81**
Non-reassuring fetal status	7 (28)	7 (25)		
Failure to progress	18 (72)	21 (75)		
Postpartum outcomes				
Delivery blood loss (ml)	300 [250–500]	350 [250–500]		0.95***
Postpartum hemorrhage (≥500 ml)	12 (29)	12 (27)	1.0 (0.5–2.1)	0.89**
Induction to discharge interval (days)	3.3 ± 0.9	3.4 ± 1.2		0.66*
Maternal satisfaction (induction to birth)	7 [6–8]	7 [7–8]^4^		0.97***
Fever^b^	2 (5)	1 (2)	2.1 (0.2–22.3)	0.53**
**Neonatal outcomes**				
Birth weight (kg)	2.94 ± 0.43	2.95 ± 0.45		0.87*
Apgar score at 5 min	10 [10–10]	10 [10–10]		0.13***
Apgar score at 5 min < = 6	0 (0)	0 (0)		
Apgar score at 1 min	9 [9–9]	9 [9–9]		0.27***
Apgar score at 1 min ≤ 4	0 (0)	0 (0)		
Admission to neonatal unit^c^	5 (12)	6 (14)	0.9 (0.3–2.6)	0.81**
*Indication for admission*				
Transient tachypnea	1 (25)	1 (17)		
Severe neonatal jaundice	1 (25)			
Presumed sepsis	1 (25)	1 (17)		
Observe for heart block		1 (17)		
Hypoglycemia		1 (17)		
Grunting	1 (25)			
Congenital pneumonia	1 (25)			
Mild bilateral ventriculomegaly		1 (17)		
Meconium aspiration syndrome		1 (17)		
Umbilical cord artery blood pH	7.3 ± 0.06	7.3 ± 0.06		0.19*
pH ≤ 7.1	0 (0)	0(0)		
Umbilical cord artery blood base excess	−4.7 ± 2.9	−5.1 ± 3.2		0.50*
Base excess ≤ −8	6 (14)	7 (16)		0.84**

Data are represented as mean ± standard deviation, number (%) or median [interquartile range]. * represents analyses by *t* test for means, ** represents Chi Square test for categorical data and *** represents Mann-Whitney *U* test for ordinal data

^a^Operative vaginal includes forceps and vacuum. For the digital arm, forceps delivery n = 2 while for the speculum arm, forceps delivery n = 1, vacuum delivery *n* = 3

^b^Fever is temperature greater than or equal to 38 degrees is recorded intrapartum or 1 day postpartum

^c^This includes special care nursery, neonatal intensive care unit and pediatric wards

### Sensitivity analysis

Post hoc, with insertion duration set as 6 min instead of the arbitrary 10 min penalty for failure (both failures were in the digital arm), mean (with standard deviation) insertion duration were 2.52 ± 1.15 vs 2.25 ± 0.55 min *p* = 0.17 in digital and speculum arm respectively; the point estimate continued to favor speculum insertion. Similarly assigning a pain score of 0 instead of 10 for failure, median [interquartile range] were 3 [1–5] vs. 3 [2–5] *p* = 0.69 in digital and speculum arm respectively.

## Discussion

For the primary outcomes of insertion duration, pain and failure, there was no significant difference across the trial arms.

Two of our main findings were in sharp contrast to Jonsson [6] who showed a significantly shorter insertion duration and less procedure related pain in favor of digital insertion. Their insertion success rate (18F Foley) catheter was 100% across both their arms but their trial arms comprised 33–48% multiparas and their inclusion criteria was only for women with Bishop score 3 to 5 compared to our exclusively nulliparas trial population (43% of our participants had Bishop Score ≤ 2); nevertheless our insertion failure rate for digital arm 2/42 (5%; 95% CI 0.5–16.7%) vs speculum arm 0/44 (0%; 95% CI 0.0–9.6%) were broadly comparable to their universal 0/21 (0 95% CI 0–18.2%) insertion failure rate.

The high insertion success rate in our digital arm albeit in lithotomy position (as also in Jonsson [6]) and coupled with their findings, supported digital insertion of the Foley catheter as a first-line insertion technique even in nulliparas with unripe cervixes.

Of note when compared to Jonsson [6] in terms of their superior finding for digital insertion procedure related pain score, their digital arm median pain score was very similar to our digital arm (3 vs 3.5) whereas the pain score of their speculum arm appeared to be higher than ours (5 vs 3). It is plausible that as their population contained multiparas, speculum visualization of the cervix through a more lax paras vagina might have required greater manipulation and pressure to the vaginal walls, hence resulted in more discomfort and took longer to accomplish whereas digital insertion through a more capacious multiparas vagina could have been easier and better tolerated. It was also plausible that term women at IoL at our center tolerated speculum examination better than digital examination [7].

In a 2018 trial report, comparing latex vs silicone 18F Foley catheter insertion (by speculum) the silicone catheter is associated with a higher rate of accidental rupture of membranes but a lower rate of insertion failure. In their nulliparas subpopulation, insertion failure rates were 8.6% vs 3.2% respectively with a significantly higher failure rate with the latex catheter [15]. The 8.6% latex catheter insertion failure rate is broadly similar to our rate 0/44 (0 95% CI 0–9.6%) in the speculum arm where we exclusively used the more pliable 16F silicone-coated latex catheter.

In 2016 trial report comparing stylet vs non-stylet 22F Foley catheters inserted digitally, when the data for their nulliparas subpopulation was considered, insertion duration were median 1.78 [IQR 1.23–2.46] vs 2.08 [1.33–3.38] minutes, procedure related pain score mean 4.82 95% CI 3.56–6.08 vs 4.52 95% CI 3.51–5.53 and failed insertion rates of 4/28 (14.3%) vs 4/30 (13.3%) [16]. In our digital insertion arm (with unmodified 16F silicone-coated latex Foley catheter) insertion duration was mean (standard deviation) 2.72 ± 1.85 min, procedure related mean pain score 3.6 ± 2.4 and failure rate 2/42 (5 95% CI 0.5–16.7%). In comparison to their non-stylet catheter arm, our results were broadly comparable but on point estimates, their insertion duration was shorter but their pain score and insertion failure rate were somewhat higher.

Downstream secondary outcomes were very similar in both our trial arms. This was to be expected as technique of catheter insertion was not anticipated to impact on labor or delivery performance. Our Cesarean section rates (60–64%) were similarly high across trial arms; our study population comprised nulliparas with unripe cervixes with a substantial proportion (47.7–57.1%) of IoL indicated by ‘non-reassuring fetal status’ who were at higher risk of Cesarean delivery [8].

In nulliparas with unripe cervixes at IoL, digital compared to speculum insertion of the Foley catheter in the lithotomy position resulted in similar insertion duration, pain and failure. The point estimates for these insertion outcomes favored speculum aided insertion but the small differences of < 30 s in insertion duration, ½ point difference in an 11-point pain scale and failure rates of 0% vs. 5% in favor of speculum insertion were suggestive of clinical equivalence or non-inferiority for digital insertion. Hence digital insertion which required less equipment and consumables and with better potential for dorsal recumbent position insertion, could be preferable for the trained care provider and could be the insertion technique to be learnt by trainees assuming a similar learning curve and skill attainment potential.

### Strengths and limitations

Our strengths was a trial powered at 90% based on significant pilot data from the solitary trial by Jonsson [6]. Our sample size was twice as large and exclusively on the technically more challenging nulliparas with unripe cervixes. Our insertion protocol was fully described and conducted under tightly controlled experimental conditions. We had no post-randomization drop-outs – data was complete. HMC was the sole and experienced inserter in our trial which provided for a strong efficacy evaluation of the insertion techniques.

As to limitations, our sample size might be too small to detect small differences in insertion duration and procedure related pain but given the close point estimates for these outcomes, we assert near clinical efficacy equivalence. Insertion success rates were very high in both our trial arms (95–100%) such that our assumptions of 90% vs 60% appeared too pessimistic toward digital insertion success and rendered our sample size underpowered for this outcome. As we had a single inserter our finding was potentially less generalizable. Based on our data, a trial predicated on insertion duration with confidence interval of 95% and power at 80% will require 266 women (133 in each arm). A larger multicenter trial could be conducted in the future to better determine the preferable method.

## Conclusion

Digital insertion had similar insertion performance characteristics compared to speculum insertion of the Foley catheter for IoL even in nulliparas with unripe cervixes. Digital insertion required less equipment, consumables and had better potential for dorsal recumbent positioning, so could be the insertion technique of choice if the care provider is equally adept or the insertion technique to acquire for trainees.

## Supplementary information

**Additional file 1.**

## Data Availability

All data generated or analysed during this study are included in this published article and the datasets used are available from the corresponding author on reasonable request.

## Abbreviations

IoL: Induction of labor
NHS: National Health Service
VNRS: Visual numerical rating scale
