# Supplementary material for: Speculum versus digital insertion of Foley catheter for induction of labor in Nulliparas with unripe cervix: a randomized controlled trial
Source: BMC Pregnancy Childbirth. 2020 May 29;20:330. doi: 10.1186/s12884-020-03029-0 (PMC7257160; doi:10.1186/s12884-020-03029-0)
Supplement: Supplementary file 1 — Additional file 1. [file 12884_2020_3029_MOESM1_ESM.docx]

APPENDIX 1:

PubMed search was done on 1st July 2017, using the keywords‘Foley’, induction of ‘labour’ or‘labor,‘randomised’or‘randomized’ and ‘trial’, retrieved 120 publications of which 99 were clinical trial reports. We were able to obtain 77 publications in full text to ascertain method of insertion. Two insertion methods were described: speculum and digital insertion. In 47 of the 77 reports where insertion method was specified, 40 (85%) specified exclusive speculum insertion, 6 (12.8%) permitted either speculum or digital insertion, and only 1(2.1%) used digital insertion exclusively. Speculum insertion is predominantly favored in the literature.

| No | Author | Year | Country | Foley Size/ balloon inflation volume | n | f | Technique |
| --- | --- | --- | --- | --- | --- | --- | --- |
| 1 | Forgie et al | 2016 | US | 22F/ 50mL | 123 | 16 | Digital |
| 2 | Roni Levy | 2003 | Israel | *30mL or 80mL | 205 | 0 | Speculum |
| 3 | Levy et al | 2002 | Israel | *60mL | 211 | 0 | Speculum |
| 4 | Filho et al | 2010 | Brazil | 14F/ 30mL | 121 | 0 | Speculum |
| 5 | Niromanesh et al | 2003 | Iran | 14F/ 30mL | 45 | 0 | Speculum |
| 6 | Sciscione et al | 1998 | U.S | 14F/ 30mL | 77 | 0 | Speculum |
| 7 | Gibson K.S et al | 2013 | U.S | 14F/ 30mL | 197 | 6 | Speculum |
| 8 | Jozwiak et al | 2011 | Netherland | 16-18F/ 30mL | 412 | 13 | Speculum |
| 9 | Husain et al | 2016 | Pakistan | 16-18F/ 30mL | 169 | 5 | Speculum |
| 10 | Henry et al | 2013 | Australia | 16F/ 30mL | 50 | 0 | Speculum |
| 11 | Ugwu et al | 2013 | Nigeria | 16F/ 30mL | 50 | 0 | Speculum |
| 12 | Chung et al | 2003 | US | 16F/ 30mL | 54 | 4 | Speculum |
| 13 | Abramovici et al | 1999 | US | 16F/ 30mL | 77 | 0 | Speculum |
| 14 | Chavakula et al | 2015 | India | 16F/ 30mL | 54 | 0 | Speculum |
| 15 | Ning Gu et al | 2015 | China | 16F/ 30mL or 80mL | 504 | 2 | Speculum |
| 16 | Manish et al | 2016 | India | 16F/ 30mL or 80mL | 77 | 0 | Speculum |
| 17 | Policiano et al | 2017 | Portugal | 16F/ 40mL | 201 | 0 | Speculum |
| 18 | El-Khayat et al | 2014 | Egypt | 16F/ 60mL | 200 | 0 | Speculum |
| 19 | Owolabi et al | 2005 | Nigeria | 18F/ 30mL | 60 | 0 | Speculum |
| 20 | Onge et al | 1994 | Canada | 18F/ 30mL | 36 | 0 | Speculum |
| 21 | Fitzpatrick et al | 2012 | US | 18F/ 30mL | 136 | 19 | Speculum |
| 22 | Sharma et al | 2014 | US | 18F/ 30mL | 80 | 5 | Speculum |
| 23 | Ahmed et al | 2016 | Egypt | 18F/ 50mL | 39 | 2 | Speculum |
| 24 | Cromi et al | 2006 | Italy | 18F/ 50mL | 607 | 5 | Speculum |
| 25 | Cromi et al | 2010 | Italy | 18F/ 50mL | 131 | 1 | Speculum |
| 26 | Al-Taani MI | 2004 | Iran | 18F/ 50mL | 72 | 0 | Speculum |
| 27 | Mizrachi et al | 2016 | Berlin | 22F/ 80mL | 173 | 0 | Speculum |
| 28 | Barrilleaux et al | 2002 | US | 24F/ 50mL | 223 | 0 | Speculum |
| 29 | Hill et al | 2009 | US | 24F/ 50mL | 114 | 4 | Speculum |
| 30 | Barkai et al | 1997 | Israel | 26F/ 30mL | 48 | 0 | Speculum |
| 31 | Maslovitz et al | 2009 | Israel | 26F/ 50mL | 1083 | 19 | Speculum |
| 32 | Aduloju et al | 2016 | New Zealand | 16F/ 30mL | 70 | 0 | Speculum /+ sponge forceps |
| 33 | Dalui et al | 2003 | India | 16F/ 30mL | 50 | 0 | Speculum & sponge forceps |
| 34 | Mei-Dan et al | 2011 | US | 16F/ 30mL | 88 | 1 | Speculum & sponge forceps |
| 35 | Adeniji et al | 2005 | Nigeria | 16F/ 50mL | 96 | 0 | Speculum & sponge forceps |
| 36 | El Khouly | 2016 | Egypt | 18F/ 30mL | 72 | 0 | Speculum & sponge forceps |
| 37 | M. Kandil et al | 2012 | Egypt | 18F/ 30mL | 50 | 0 | Speculum& sponge forceps |
| 38 | Thomas et al | 1986 | UK | 18F/ 30mL | 32 | 0 | Speculum & sponge forceps |
| 39 | Guinn et al | 2003 | US | 22F/ 30mL | 100 | 13 | Speculum, if failed - Speculum & sponge forceps |
| 40 | Pettker et al | 2008 | US | 20F/ 30mL | 200 | 0 | Speculum/ +sponge forceps |
| 41 | Karjane et al | 2006 | US | 30F/ 50mL | 142 | 3 | Speculum, if failed - digital |
| 42 | Amorosa et al | 2017 | Newland | 16F/ 30mL | 62 | 0 | Speculum & sponge forceps/ Digital |
| 43 | Eikelder et al | 2016 | Netherland | 16-18F/ 30mL | 921 | 49 | Speculum or digital |
| 44 | Shuchita et al | 2017 | US | 18F/ 30mL | 602 | 4 | Speculum or Digital |
| 45 | Levine et al | 2016 | US | 18F/ 30mL | 248 | 9 | Speculum or Digital |
| 46 | Jonsson et al | 2011 | Sweden | 18F/ 50mL | 42 | 0 | Speculum or Digital |
| 47 | Carbone et al | 2013 | US | *60ml | 123 | 0 | Speculum or Digital |
| 48 | Onah H.E | 2002 | Nigeria | *30mL | 30 | 0 | Unspecified |
| 49 | Sandberg et al | 2017 | Netherland | *30mL or 80mL | 174 | 0 | Unspecified |
| 50 | Surita et al | 2004 | Brazil | 14F/ 30mL | 70 | 0 | Unspecified |
| 51 | Pennell et al | 2009 | Australia | 16F/ 30mL | 109 | 1 | Unspecified |
| 52 | Sciscione et al | 2003 | Newark | 16F/ 30mL | 63 | 0 | Unspecified |
| 53 | Edward et al | 2014 | US | 16F/ 30mL | 185 | 0 | Unspecified |
| 54 | James et al | 1994 | India | 16F/ 30mL | 187 | 0 | Unspecified |
| 55 | Patabendige et al | 2017 | Sri Lanka | 16F/ 50mL | 56 | 0 | Unspecified |
| 56 | Connolly et al | 2017 | US | 16F/ 60mL | 141 | 0 | Unspecified |
| 57 | M. Kashanian | 2005 | Iran | 16F/ Unspecified | 100 | 0 | Unspecified |
| 58 | Ziyaudin et al | 2013 | India | 16F/30mL | 35 | 0 | Unspecified |
| 59 | Ducarme et al | 2015 | France | 16F/30mL | 255 | 0 | Unspecified |
| 60 | Dahiya K et al | 2012 | India | 16F/50mL | 50 | 0 | Unspecified |
| 61 | Tabowei et al | 2003 | Nigeria | 16F/50mL | 61 | 0 | Unspecified |
| 62 | Culver et al | 2004 | US | 18F/ 30mL | 83 | 0 | Unspecified |
| 63 | Liu et al | 1998 | Taiwan | 18F/ 30mL | 32 | 4 | Unspecified |
| 64 | Afolabi et al | 2005 | Nigeria | 18F/ 30mL | 50 | 0 | Unspecified |
| 65 | Delaney et al | 2010 | US | 18F/ 30mL or 60mL | 192 | 0 | Unspecified |
| 66 | Gonsalves et al | 2016 | Oman | 18F/ 30mL to 60mL | 68 | 0 | Unspecified |
| 67 | Bujold et al | 2004 | US | 18F/ 50mL | 255 | 0 | Unspecified |
| 68 | Gelisen et al | 2004 | Turkey | 18F/ 50mL | 100 | 8 | Unspecified |
| 69 | Fatemeh et al | 2012 | Iran | 18F/ 50mL | 59 | 0 | Unspecified |
| 70 | Mullin et al | 2002 | US | 18F/ 60mL | 100 | 0 | Unspecified |
| 71 | Moini et al | 2003 | Iran | 22F/ 30mL | 35 | 0 | Unspecified |
| 72 | Ghanaie et al | 2013 | Iran | 22F/ 30mL | 240 | 2 | Unspecified |
| 73 | Kruit et al | 2015 | Finland | 22F/ 30mL to 60mL | 432 | 0 | Unspecified |
| 74 | Kruit et al | 2017 | Finland | 22F/ 50mL | 361 | 0 | Unspecified |
| 75 | Hemlin et al | 1998 | Sweden | 24F/ 30mL | 43 | 0 | Unspecified |
| 76 | Kashanian et al | 2008 | Iran | 24F/ 30mL or 80mL | 180 | 0 | Unspecified |
| 77 | Perry K. G et al | 1997 | US | 24F/ 50mL | 65 | 0 | Unspecified |
|  | n : Number of participants in the trial  f : Number of failed insertions mentioned  *: Gauge of Foley catheter used in trial was not specified | | | | | | |

References:

1. Forgie, M. M., Greer, D. M., Kram, J. J., Wyst, K. B., Salvo, N. P., & Siddiqui, D. S. (2016). Foley catheter placement for induction of labor with or without stylette: A randomized clinical trial. American Journal of Obstetrics and Gynecology, 214(3). doi:10.1016/j.ajog.2015.12.043
2. Levy R, Kanengiser B, Furman B, Ben Arie A, Brown D, Hagay ZJ. A randomized trial comparing a 30-mL and an 80-mL Foley catheter balloon for preinduction cervical ripening. Obstet Gynecol. 2004; 191:1632–1636.
3. Levy R, Ferber A, Ben-Arie A, Paz B, Hazan Y, Blickstein I, Hagay ZJ. [A randomised comparison of early versus late amniotomy following cervical ripening with a Foley catheter.](https://www.ncbi.nlm.nih.gov/pubmed/11888099) BJOG. 2002 Feb;109(2):168-72.
4. Filho OB, Albuquerque RM, Cecatti JG. A randomized controlled trial comparing vaginal misoprostol versus Foley catheter plus oxytocin for labor induction. Acta Obstet Gynecol Scand 2010;89:1045–52
5. Niromanesh S, Mosavi-Jarrahi A, Samkhaniani F. Intracervical Foley catheter balloon vs. prostaglandin in preinduction cervical ripening. Int J Gynaecol Obstet 2003;81:23–7
6. Sciscione AC, McCullough H, Manley JS, Shlossman PA, Pollock M, Colmorgen GH. A prospective, randomized comparison of Foley catheter insertion versus intracervical prostaglandin E2 gel for preinduction cervical ripening. Am J Obstet Gynecol 1999;180:55–60
7. Gibson KS, Mercer BM, Louis JM. Inner thigh taping vs traction for cervical ripening with a Foley catheter: a randomized controlled trial. Am J Obstet Gynecol 2013;209:272.e1-7
8. Jozwiak M, Bloemenkamp KW, Kelly AJ, Mol BW, Irion O, Boulvain M. Mechanical methods for induction of labour. Cochrane Database Syst Rev 2012;3:CD001233.
9. Husain S, Husain S, Izhar R. Oral misoprostol alone versus oral misoprostol and Foley's catheter for induction of labor: A randomized controlled trial. J Obstet Gynaecol Res. 2017 Aug;43(8):1270-1277. doi: 10.1111/jog.13354.
10. Henry A, Madan A, Reid R, Tracy S, Austin K, Welsh A, et al Outpatient Foley catheter versus inpatient prostaglandin E2 gel for induction of labour: a randomised trial. BMC Pregnancy Childbirth 2013; 13 25
11. Ugwu EO, Onah HE, Obi SN, Dim CC, Okezie OA, Chigbu CO, et al. Effect of the Foley catheter and synchronous low dose misoprostol administration on cervical ripening: a randomised controlled trial. J Obstet Gynaecol 2013;33(6):572–7
12. Chung JH, Huang WH, Rumney PJ, Garite TJ, Nageotte MP. A prospective randomized controlled trial that compared misoprostol, Foley catheter, and combination misoprostol-Foley catheter for labor induction. Am J Obstet Gynecol 2003;189(4): 1031–5.
13. Chung JH, Huang WH, Rumney PJ, Garite TJ, Nageotte MP. A prospective randomized controlled trial that compared misoprostol, Foley catheter, and combination misoprostol-Foley catheter for labor induction. Am J Obstet Gynecol 2003;189(4): 1031–5.
14. Chavakula PR, Benjamin SJ, Abraham A, Londhe V, Jeyaseelan V, Mathews JE. Misoprostol versus Foley catheter insertion for induction of labor in pregnancies affected by fetal growth restriction. Int J Gynaecol Obstet. 2015 May;129(2):152-5.
15. Ning Gu, Tong Ru, Zhiqun Wang, Yimin Dai, Mingming Zheng, Biyun Xu, Yali Hu. Foley Catheter for Induction of Labor at Term. An Open-Label, Randomized Controlled Trial. PLos ONE 10(8):e0136856.
16. Manish P, Rathore S, Benjamin SJ3, Abraham A2, Jeyaseelan V4, Mathews JE. A randomised controlled trial comparing 30 mL and 80 mL in Foley catheter for induction of labour after previous Caesarean section. Trop Doct. 2016 Oct;46(4):205-211.
17. Catarina Policiano, Mariana Pimenta, Diana Martins, Nuno Clode. Efficacy and safety of Foley Catheter Ballon for Cervix Priming in Term Pregnancy. Acta Med Port 2017 Apr;30(4):281-284
18. El-Khayat W, Alelaiw H, El-kateb A, Elsemary A. [Comparing vaginal misoprostol versus Foley catheter plus vaginal isosorbide mononitrate for labor induction.](https://www.ncbi.nlm.nih.gov/pubmed/25694257) J Matern Fetal Neonatal Med. 2016;29(3):487-92.
19. Owolabi AT, Kuti O, Ogunlola IO. [Randomised trial of intravaginal misoprostol and intracervical Foley catheter for cervical ripening and induction of labour.](https://www.ncbi.nlm.nih.gov/pubmed/16234141) J Obstet Gynaecol. 2005 Aug;25(6):565-8.
20. St Onge RD, Connors GT. [Preinduction cervical ripening: a comparison of intracervical prostaglandin E2 gel versus the Foley catheter.](https://www.ncbi.nlm.nih.gov/pubmed/7856707) Am J Obstet Gynecol. 1995 Feb;172(2 Pt 1):687-90
21. Fitzpatrick CB, Grotegut CA, Bishop TS, Canzoneri BJ, Heine RP, Swamy GK. [Cervical ripening with foley balloon plus fixed versus incremental low-dose oxytocin: a randomized controlled trial.](https://www.ncbi.nlm.nih.gov/pubmed/21793769) J Matern Fetal Neonatal Med. 2012 Jul;25(7):1006-10. doi: 10.3109/14767058.2011.607522.’
22. Sharma KJ, Grubbs BH, Mullin PM, Opper N, Lee RH. [Labor induction utilizing the Foley balloon: a randomized trial comparing standard placement versus immediate removal.](https://www.ncbi.nlm.nih.gov/pubmed/25569680) J Perinatol. 2015 Jun;35(6):390-5. doi: 10.1038/jp.2014.229.
23. Sayed Ahmed, W. A., Ibrahim, Z. M., Ashor, O. E., Mohamed, M. L., Ahmed, M. R., and Elshahat, A. M. (2016) Use of the Foley catheter versus a double balloon cervical ripening catheter in pre-induction cervical ripening in postdate primigravidae. J. Obstet. Gynaecol. Res., 42: 1489–1494. doi: [10.1111/jog.13086](http://dx.doi.org/10.1111/jog.13086).
24. Cromi A, Ghezzi F, Tomera S, Ucella S, Lischetti B, Bolis P.F. Cervical ripening with the Foley catheter. International Journal of Gynaecology and Obstetrics (2007)97,105-109.
25. Cromi A, Ghezzi F, Agosti M, Serati M, Uccella S, Arlant V, et al. Is transcervical Foley [catheter](https://www.ncbi.nlm.nih.gov/pubmedhealth/PMHT0022169) actually slower than prostaglandins in ripening the [cervix](https://www.ncbi.nlm.nih.gov/pubmedhealth/PMHT0021895)? A [randomized study](https://www.ncbi.nlm.nih.gov/pubmedhealth/PMHT0025811). Am J Obstet Gynecol 2011;204:338.e1–7.
26. Al-Taani MI. comparison of prostaglandin E_2_ tablets or Foley catheter for labour induction in grand multiparas. Easwt Mediten Health J 2004;200410:547-53.
27. Mizrachi Y, Levy M, Bar J, Kovo M. [Induction of labor in nulliparous women with unfavorable cervix: a comparison of Foley catheter and vaginal prostaglandin E2.](https://www.ncbi.nlm.nih.gov/pubmed/26837386) Arch Gynecol Obstet. 2016 Oct;294(4):725-30. doi: 10.1007/s00404-016-4026-9.
28. P.Scott Barrilleaux, James A. Bofill, Dom A. Terrone, Everett F. Magann, Warren L. May, John C. Morrison. Cervical ripening and induction of labor with misoprostol, dinoprostone gel, and a foley catheter: A randomized trial of 3 techniques. Am J Obstet Gynecol 2002;186;1124–1129.
29. Hill JB, Thigpen BD, Bofill JA, Magann E, Moore LE, Martin JN Jr. A randomized clinical trial comparing vaginal misoprostol versus cervical Foley plus oral misoprostol for cervical ripening and labor induction. Am J Perinatol 2009; 26:33–8
30. Barkai G, Cohen SB, Kees S, Lusky A, Margalit V, Mashiach S, Schiff E.[Induction of labor with use of a Foley catheter and extraamniotic corticosteroids.](https://www.ncbi.nlm.nih.gov/pubmed/9396910) Am J Obstet Gynecol. 1997 Nov;177(5):1145-8.
31. Maslovitz S, Lessing JB, Many A. [Complications of trans-cervical Foley catheter for labor induction among 1,083 women.](https://www.ncbi.nlm.nih.gov/pubmed/19488776) Arch Gynecol Obstet. 2010 Mar;281(3):473-7. doi: 10.1007/s00404-009-1136-7. Epub 2009 Jun 2.
32. Aduloju OP, Akintayo AA, Adanikin AI et al. Combined Foley’s catheter with vaginal misoprostol for pre-induction cervical ripening: A randomised controlled trial. Aust N Z J Obstet Gynaecol. 2016;
33. Dalui R, Suri V, Ray P, Gupta I. Comparison of extraamniotic Foley Catheter and intracervical prostaglandin E gel for preinduction cervical ripening. Acta Obstet Gynaecol Scand 2005;84:362-7
34. Mei-Dan E, Walfisch A, Suarez-Easton S, Hallak M. [Comparison of two mechanical devices for cervical ripening: a prospective quasi-randomized trial](https://www.ncbi.nlm.nih.gov/pubmed/21806490). J Matern Fetal Neonatal Med. 2012 Jun;25(6):723-7
35. Adeniji OA, Oladokun A, Olayemi O, Adeniji OI, Odukogbe AA, Ogunbode O, et al. Pre-induction cervical ripening: transcervical foley catheter versus intravaginal misoprostol. J Obstet Gynaecol 2005;25(2):134–9.
36. El Khouly NI. A prospective randomized trial comparing Foley catheter, oxytocin, and combination Foley catheter-oxytocin for labour induction with unfavourable cervix. J Obstet Gynaecol. 2017 Apr;37(3):309-314. doi: 10.1080/01443615.2016.1239075
37. Kandil M, Emarh M, Sayyed T, Masood A. [Foley catheter versus intra-vaginal misoprostol for induction of labor in post-term gestations.](https://www.ncbi.nlm.nih.gov/pubmed/22434058) Arch Gynecol Obstet. 2012 Aug;286(2):303-7. doi: 10.1007/s00404-012-2292-8
38. Thomas IL, Chenoweth JN, Tronc GN, Johnson IR. Preparation for induction of labour of the unfavourable cervix with Foley catheter compared with vaginal prostaglandin. Aust N Z J Obstet Gynaecol. 1986 Feb;26(1):30-5.
39. Guinn DA, Davies JK, Jones RO, Sullivan L, Wolf D. Labor induction in women with an unfavorable Bishop score: randomized controlled trial of intrauterine Foley catheter with concurrent oxytocin infusion versus Foley catheter with extra-amniotic saline infusion with concurrent oxytocin infusion. Am J Obstet Gynecol 2004;191(1): 225–9.
40. Pettker CM, Pocock SB, Smok DP, Lee SM, Devine PC. [Transcervical Foley catheter with and without oxytocin for cervical ripening: a randomized controlled trial.](https://www.ncbi.nlm.nih.gov/pubmed/18515515) Obstet Gynecol. 2008 Jun;111(6):1320-6. doi: 10.1097/AOG.0b013e31817615a0.
41. Karjane NW, Brock EL, Walsh SW. [Induction of labor using a foley balloon, with and without extra-amniotic saline infusion.](https://www.ncbi.nlm.nih.gov/pubmed/16449106) Obstet Gynecol. 2006 Feb;107(2 Pt 1):234-9.
42. Amorosa JMH, Stone J, Factor SH, Booker W, Newland M, Bianco A. A randomized trial of Foley Bulb for Labor Induction in Premature Rupture of Membranes in Nulliparas (FLIP). Am J Obstet Gynecol. 2017 May 4. pii: S0002-9378(17)30568-9. doi: 10.1016/j.ajog.2017.04.038
43. Ten Eikelder M, van Baaren GJ, Oude Rengerink K, Jozwiak M, de Leeuw JW, Kleiverda G, Evers I, de Boer K, Brons J, Bloemenkamp K, Mol BW. Comparing induction of labour with oral misoprostol or Foley catheter at term: cost-effectiveness analysis of a randomised controlled multi-centre non-inferiority trial. Comparing induction of labour with oral misoprostol or Foley catheter at term: cost-effectiveness analysis of a randomised controlled multi-centre non-inferiority trial.
44. Shuchita Mundle, Hillary Bracken, Vaishali Khedikar, Jayashree Mulik, Brian Faragher, Thomas Easterling, Simon Leigh, Paul Granby, Alan Haycox, Mark A Turner, Zarko Alfirevic, Beverly Winikoff, Andrew D Weeks. Foley catheterisation versus oral misoprostol for induction of labour in hypertensive women in India (INFORM): a multicentre, open-label, randomised controlled trial. The Lancet, 2017
45. Levine LD, Downes KL, Elovitz MA, Parry S, Sammel MD, Srinivas SK. [Mechanical and Pharmacologic Methods of Labor Induction: A Randomized Controlled Trial.](https://www.ncbi.nlm.nih.gov/pubmed/27824758) Obstet Gynecol. 2016 Dec;128(6):1357-1364.
46. Jonsson M, Hellgren C, Wiberg-Itzel E, Akerud H. [Assessment of pain in women randomly allocated to speculum or digital insertion of the Foley catheter for induction of labor.](https://www.ncbi.nlm.nih.gov/pubmed/21615714) Acta Obstet Gynecol Scand. 2011 Sep;90(9):997-1004. doi: 10.1111/j.1600-0412.2011.01197
47. Carbone JF, Tuuli MG, Fogertey PJ, Roehl KA, Macones GA. Combination of Foley bulb and vaginal misoprostol compared with vaginal misoprostol alone for cervical ripening and labor induction: a randomized controlled trial. Obstet Gynecol. 2013 Feb;121(2 Pt 1):247-52. doi: http://10.1097/AOG.0b013e31827e5dca.
48. Onah HE. [Effect of the Foley catheter and synchronous oxytocin administration on cervical ripening.](https://www.ncbi.nlm.nih.gov/pubmed/12834946) Int J Gynaecol Obstet. 2003 Jul;82(1):71-2
49. Sandberg EM, Schepers EM, Sitter RL, Huisman CM, Wijngaarden WJ. Foley catheter for induction of labour filled with 30mL or 60mL: A randomized controlled trial. [Foley catheter for induction of labour filled with 30mL or 60mL: A randomized controlled trial.](https://www.ncbi.nlm.nih.gov/pubmed/28260689)
50. Surita FG, Cecatti JG, Parpinelli MA, Krupa F, Pinto E Silva JL. [Hyaluronidase](https://www.ncbi.nlm.nih.gov/pubmedhealth/PMHT0010577) versus Foley [catheter](https://www.ncbi.nlm.nih.gov/pubmedhealth/PMHT0022169) for cervical ripening in high-risk term and post term [pregnancies](https://www.ncbi.nlm.nih.gov/pubmedhealth/PMHT0023077). Int J Gynaecol Obstet 2005;88:258–64.
51. Pennell CE, Henderson JJ, O’Neill MJ, McCleery S, Doherty DA, Dickinson JE. Induction of labour in nulliparous women with an unfavourable cervix: a randomised controlled trial comparing double and single balloon catheters and PGE2 gel. BJOG. 2009; 116: 1443-1452.
52. Sciscione AC, Nguyen L, Manley J, Pollock M, Maas B, Colmorgen G. A randomized comparison of transcervical Foley catheter to intravaginal misoprostol for preinduction cervical ripening. Obstet Gynecol 2001;97(4):603–7.
53. Edwards RK, Szychowski JM, Berger JL, Petersen M, Ingersoll M, Bodea-Braescu AV, Lin MG. [Foley catheter compared with the controlled-release dinoprostone insert: a randomized controlled trial.](https://www.ncbi.nlm.nih.gov/pubmed/24807327) Obstet Gynecol. 2014 Jun;123(6):1280-7. doi: 10.1097/AOG.0000000000000238.
54. C. James, A. Peedicayil, L. seshadri. Use of Foley catheter as cervical ripening agent prior to induction of labour. International Journal of gynaecology & Obstetrics 57(1994)229-232.
55. Patabendige M, Jayawardane. Foley catheter for cervical priming in induction of labour at University Obstetrics Unit, Colombo, Sri Lanka: a clinical audit with a patient satisfaction survey. BMC nRes Notes (2017) 10:155
56. Connolly KA, Kohari KS, Rekawek P, Smilen BS, Miller MR, Moshier E, Factor SH, Stone JL, Bianco AT. [A randomized trial of Foley balloon induction of labor trial in nulliparas (FIAT-N).](https://www.ncbi.nlm.nih.gov/pubmed/27018464) Am J Obstet Gynecol. 2016 Sep;215(3):392.e1-6. doi: 10.1016/j.ajog.2016.03.034.
57. Kashanian M, Akbarian AR, Fekrat M. Cervical ripening and induction of labor with intravaginal misoprostol and Foley catheter cervical traction. Int J Gynecol Obstet 2006;92(1):79–80.
58. Ziyauddin F, Hakim S, Beriwal S. The transcervical foley catheter versus the vaginal prostaglandin e2 gel in the induction of labour in a previous one caesarean section - a clinical study. J Clin Diagn Res. 2013 Jan;7(1):140-3. doi: 10.7860/JCDR/2012/5003.2689.
59. Ducarme G, Grange J, Vital M.: Expansion dilatation balloons for cervical ripening in obstetric practice. J Gynecol Obstet Biol Reprod (Paris). 2016 Feb;45(2):112-9. doi: 10.1016/j.jgyn.2015.11.007.
60. Krishna Dahiya, Kanika Malik, Archit Dahiya, Smiti Nanda: Comparison of the Efficacy of Foley Catheter Balloon with Dinoprostone Gel for Cervical Ripening at Term. International Journal of Clinical Medicine, 2012, 3, 527-531. DOI: 10.4236/ijcm.2012.36095
61. Tabowei TO1, Oboro VO. Low dose intravaginal misoprostol versus intracervical baloon catheter for pre-induction cervical ripening. East Afr Med J. 2003 Feb;80(2):91-4.
62. Culver J, Strauss RA, Brody S, Dorman K, Timlin S, McMahon MJ. [A randomized trial comparing vaginal misoprostol versus Foley catheter with concurrent oxytocin for labor induction in nulliparous women.](https://www.ncbi.nlm.nih.gov/pubmed/15085496) Am J Perinatol. 2004 Apr;21(3):139-46.
63. Liu HS, Chang YK, Chu TY, Yu MH, Chen WH.: Extra-amniotic balloon with PGE2 versus extra-ovular Foley catheter with PGF2alpha in mid-trimester pregnancy termination. Int J Gynaecol Obstet. 1998 Oct;63(1):51-4.
64. Afolabi BB, Oyeneyin OL, Ogedengbe OK.: [Intravaginal misoprostol versus Foley catheter for cervical ripening and induction of labor.](https://www.ncbi.nlm.nih.gov/pubmed/15919393) Int J Gynaecol Obstet. 2005 Jun;89(3):263-7
65. Delaney S, Shaffer BL, Cheng YW, Vargas J, Sparks TN, Paul K, Caughey AB. [Predictors of cesarean delivery in women undergoing labor induction with a Foley balloon.](https://www.ncbi.nlm.nih.gov/pubmed/25012806) J Matern Fetal Neonatal Med. 2015 Jun;28(9):1000-4. doi: 10.3109/14767058.2014.944154
66. Gonsalves H, Al-Riyami N, Al-Dughaishi T, Gowri V, Al-Azri M, Salahuddin A. Use of Intracervical Foley Catheter for Induction of Labour in Cases of Previous Caesarean Section: Experience of a single tertiary centre in Oman. Sultan Qaboos University Medical Journal. 2016;16(4):e445-e450. doi:10.18295/squmj.2016.16.04.007.
67. Bujold E, Blackwell SC, Gauthier RJ. [Cervical ripening with transcervical foley catheter and the risk of uterine rupture.](https://www.ncbi.nlm.nih.gov/pubmed/14704239) Obstet Gynecol. 2004 Jan;103(1):18-23.
68. Gelisen O, Caliskan E, Dilbaz S, Ozdas E, Dilbaz B, Ozdas E, Haberal A. [Induction of labor with three different techniques at 41 weeks of gestation or spontaneous follow-up until 42 weeks in women with definitely unfavorable cervical scores.](https://www.ncbi.nlm.nih.gov/pubmed/15925045) Eur J Obstet Gynecol Reprod Biol. 2005 Jun 1;120(2):164-9.
69. Fetemeh Vahid Roudsari F, Ayati S, Ghasemi M, Hasanzadeh Mofrad M, Shakeri MT, Farshidi F, Shahabian M. Comparison of vaginal misoprostol with foley catheter for cervical ripening and induction of labor. Iran J Pharm Res. 2011 Winter;10(1):149-54.
70. Mullin PM, House M, Paul RH, Wing DA. A comparison of vaginally administered misoprostol with extra-amniotic saline solution infusion for cervical ripening and labor induction. Am J Obstet Gynecol. 2002 Oct;187(4):847-52.
71. Moini A, Riazi K, Honar H, Hasanzadeh Z. Preinduction cervical ripening with the Foley catheter and saline infusion vs. cervical dinoprostone. Int J Gynaecol Obstet. 2003 Nov;83(2):211-3.
72. Ghanaie MM, Jafarabadi M, Milani F, Asgary SA, Karkan MF. A randomized controlled trial of foley catheter, extra-amniotic saline infusion and prostaglandin e2 suppository for labor induction. J Family Reprod Health. 2013 Jun;7(2):49-55.
73. Kruit H, Heikinheimo O, Ulander VM, Aitokallio-Tallberg A, Nupponen I, Paavonen J, Rahkonen L. Management of Foley catheter induction among nulliparous women: a retrospective study. BMC Pregnancy Childbirth. 2015 Oct 27;15:276. doi: 10.1186/s12884-015-0715-9.
74. Kruit H, Wilkman H, Tekay A, Rahkonen L. Induction of labor by Foley catheter compared with spontaneous onset of labor after previous cesarean section: a cohort study. J Perinatol. 2017 Jul;37(7):787-792. doi: 10.1038/jp.2017.50. Epub 2017 Apr 13.
75. Hemlin J, Möller B. Extraamniotic saline infusion is promising in preparing the cervix for induction of labor. Acta Obstet Gynecol Scand. 1998 Jan;77(1):45-9.
76. Kashanian M, Nazemi M, Malakzadegan A. [Comparison of 30-mL and 80-mL Foley catheter balloons and oxytocin for preinduction cervical ripening.](https://www.ncbi.nlm.nih.gov/pubmed/19232606) Int J Gynaecol Obstet. 2009 May;105(2):174-5. doi: 10.1016/j.ijgo.2009.01.005.
77. Perry, KG, Larmon, JE, May, WL, Robinette, LG, Martin, RW. Cervical ripening: a randomized comparison between intravaginal misoprostol and an intracervical balloon catheter combined with intravaginal dinoprostone. Am J Obstet Gynecol. 1998;178:1333–1340
